# Lessons learned from applying established cut-off values of questionnaires to detect somatic symptom disorders in primary care: a cross-sectional study

**DOI:** 10.3389/fpsyt.2023.1289186

**Published:** 2024-01-18

**Authors:** Victoria von Schrottenberg, Anne Toussaint, Alexander Hapfelmeier, Clara Teusen, Bernhard Riedl, Peter Henningsen, Jochen Gensichen, Antonius Schneider, Klaus Linde

**Affiliations:** ^1^Department Clinical Medicine, Institute of General Practice and Health Services Research, TUM School of Medicine and Health, Technical University of Munich, Munich, Germany; ^2^Department of Psychosomatic Medicine and Psychotherapy, University Medical Center Hamburg-Eppendorf, Hamburg, Germany; ^3^Institute of AI and Informatics in Medicine, TUM School of Medicine and Health, Technical University of Munich, Munich, Germany; ^4^Department of Psychosomatic Medicine and Psychotherapy, University Hospital, Technical University of Munich, Munich, Germany; ^5^Institute of General Practice and Family Medicine, University Hospital of the Ludwig-Maximilians-University of Munich, Munich, Germany

**Keywords:** somatic symptom disorder, primary healthcare, sensitivity and specificity, questionnaires, SSD-12, PHQ-15

## Abstract

**Introduction:**

Based on two diagnostic accuracy studies in high-prevalence settings, two distinctly different combinations of cut-off values have been recommended to identify persons at risk for somatic symptom disorder (SSD) with the combination of the Patient-Health Questionnaire-15 (PHQ-15) and the Somatic Symptom Disorder—B Criteria Scale (SSD-12). We investigated whether the reported sensitivity and specificity of both recommended cut-off combinations are transferable to primary care.

**Methods:**

In a cross-sectional study, 420 unselected adult primary care patients completed PHQ-15 and SSD-12. Patients scoring ≥9 and ≥ 23 (recommended cut-off combination #1) or ≥ 8 and ≥ 13 (recommended cut-off combination #2) were considered test-positive for SSD, respectively. To assess the validity of the reported sensitivity and specificity in different low- to high-prevalence settings, we compared correspondingly expected proportions of test positives to the proportion observed in our sample.

**Results:**

Based on combination #1, 38 participants (9%) were found to be test positive, far fewer than expected, based on the reported values for sensitivity and specificity (expected minimum frequency 30% with a true prevalence ≥1%). This can only be explained by a lower sensitivity and higher specificity in primary care. For combination #2, 98 participants (23%) were test positive, a finding consistent with a true prevalence of SSD of 15% or lower.

**Discussion:**

Our analyzes strongly suggest that the sensitivity and specificity estimates reported for combination #1 are not applicable to unselected primary care patients and that the cut-off for the SSD (≥23) is too strict. Cut-off combination #2 seems more applicable but still needs to be tested in studies that compare screening findings by questionnaires with validated diagnostic interviews as reference standards in primary care populations.

## 1 Introduction

Based on the accumulating knowledge of the pathogenesis and diagnosis of persistent somatic symptoms, the fifth edition of the Diagnostic and Statistical Manual of Mental Disorders (DSM-5) introduced major changes in the way of defining somatoform disorders in 2013 (1). The former category, “Somatoform and Related Disorders,” was replaced by a new category, i.e., “Somatic Symptom and Related Disorders” (SSD). While a medical explanation for the occurrence of somatic symptoms was an exclusion criterion for somatoform disorders in DSM-IV (2), SSD is characterized by somatic symptoms that are distressing or result in a significant disruption of daily life, regardless of their etiology (A criterion), by excessive thoughts, feelings, or behaviors regarding the somatic complaints or associated health concerns (B criterion), as well as by a persistent state (at least 6 months) of being symptomatic (C criterion). In 2022, the International Classification of Diseases, 11th Revision (ICD-11) introduced a similar new diagnostic category, i.e., Bodily Distress Disorder (3).

Given the high prevalence of somatoform disorders and medically unexplained symptoms in primary care (4), the shift to SSD is of major relevance to general practitioners. In a recent large survey, German general practitioners estimated the proportion of their patients fulfilling all the criteria of SSD to be 7.7% (5). Yet, the prevalence of SSD in primary care is still unclear as rigorous studies using semi-structured clinical interviews to make the diagnosis are lacking in this setting. Many studies estimate the prevalence of SSD using proxy diagnoses operationalized by a combination of self-report questionnaires (6).

The Patient Health Questionnaire-15 (PHQ-15) (7) and the Somatic Symptom Disorder—B Criteria Scale (SSD-12) (8, 9) are standardized, validated, and freely available patient-reported tools that can assist in assessing the A and B criteria of SSD when used in combination. Two recent studies comparing the combined results of the PHQ-15 and the SSD-12, with semi-structured diagnostic interviews for SSD as the reference standard, found good diagnostic accuracy (area under the receiver operating characteristic curve 0.77 and 0.84, respectively) (10, 11). However, study participants were recruited in a psychosomatic outpatient clinic in Germany (10) and in a variety of hospital outpatient clinics in China (11), where the prevalence of SSD was high (56 and 34%). Furthermore, the combination of recommended cut-off values and reported estimates of sensitivity and specificity differed considerably between the two studies.

In 2016, we performed a cross-sectional study in a primary care population with the primary aim of investigating differences in socio-demographic, somatic, and psychological characteristics between patients consulting their general practitioner either with or without an appointment (12). Furthermore, we investigated the psychometric characteristics and validity of the SSD-12 in primary care (9). However, as the study did not comprise a (semi-)structured diagnostic interview as the reference standard, we could neither directly investigate the prevalence of SSD nor optimal cut-offs and diagnostic accuracy. As recommendations for cut-off values have now been published (10, 11) we performed a secondary analysis of our data. Originally, we aimed to investigate differences in characteristics between patients who tested positive and negative for possible SSD. However, the two different cut-off recommendations resulted in highly discrepant proportions of test positives, which seemed hardly compatible with the sensitivity and specificity estimates reported by Toussaint et al. (10) and Cao et al. (11). In this manuscript, we describe and interpret our findings and discuss the implications of the found inconsistencies for future use of screening tools for SSD in primary care.

## 2 Materials and methods

### 2.1 Study design, procedure, and participants

The basic methods of our original study have been reported in detail previously (12). A cross-sectional study was performed between October 2015 and April 2016 in five general practices in Bavaria, Germany. Each practice was asked to invite 50 consecutive adult patients visiting the practice without a previous appointment and the following patient with an appointment to fill in a questionnaire addressing socio-demographic characteristics, the reason for encounter, urgency of seeing a physician, somatic and psychological symptoms, personality traits, and satisfaction with the practice. Patients coming only to the practice to pick up a prescription, who did not aim to see the physician, or who needed immediate emergency care were excluded, so a total of 501 patients were included.

### 2.2 Assessment with PHQ-15 and SSD-12

The PHQ-15 is a widely used instrument to identify individuals with elevated symptom burden (7). A total of 13 items document the presence and severity of common somatic symptoms on a scale from 0 (“not at all disturbed”) to 2 (“very disturbed”). Furthermore, two items address psychological symptoms. Item scores are summed up to a sum score ranging from 0 to 30. Sum scores ≥10 indicate moderate or high symptom burden. Sum scores are not calculated if more than two items are missing.

The SSD-12 includes 12 items and assesses the B criteria (four items for each of the three subscales of excessive thoughts, feelings, or behaviors regarding somatic complaints) of SSD (8). Item values range from 0 (“never”) to 4 (“very often”), resulting in a sum score range from 0 to 48. Sum scores are not calculated if more than three items (one per each subscale) are missing.

For classifying participants as “test positive” or “test negative” for SSD, we used the cut-off values recommended by Toussaint et al. (10) as combination #1: ≥9 for the PHQ-15 / ≥23 for the SSD-12; and the cut-off values recommended by Cao et al. (11) as combination #2: ≥8/≥13. Recommended values had been chosen on “efficiency values” (i.e., the total percentage of correct diagnosis, combining both true-positive and true-negative diagnosis). For combination #1, a sensitivity of 0.69 (95% confidence interval 0.62, 0.75) and specificity of 0.70 (0.62, 0.77) had been reported; for combination #2, the values were 0.68 (0.62, 0.74) and 0.80 (0.75, 0.83), respectively (10, 11).

### 2.3 Data analysis

We used SPSS 26.0 (IBM Corp., Armonk, NY, United States) for statistical analyzes. Data of participants with and without appointments were pooled as the findings for all psychometric scales, and questionnaire results were very similar in both groups, although patients without appointments tended to be younger and to have more often an acute reason for encounter (12).

The distribution of quantitative data is described by mean, median, standard deviation (SD), interquartile range (IQR), and range. Qualitative data is presented by absolute and relative frequencies. Clopper-Pearson 95 and 99% confidence intervals (95%CI and 99%CI) are used to describe the precision of estimates and to explore the value range of unknown frequencies.

To investigate how well the observed proportions of test positives fit with the sensitivity [the probability of the test in classifying correctly a truly ill person with SSD as test positive; formula: test positive / (test positive + false negative)] and specificity [the probability of the test in classifying a truly healthy person as test negative according to SSD diagnosis; formula: test negative / (true negative + false positive)] estimates reported by Toussaint et al. (10) for combination #1 and Cao et al. (11) for combination #2, we calculated 2 × 2 tables. For both cut-off combinations described above, we derived the expected number of true positives, false positives, false negatives, and true negatives assuming a wide range of eight possible “true” prevalence values (1, 5, 10, 15, 20, 25, 30, and 40%) for a sample size of 420 participants. We also used the law of total probability to determine the probability of observing a test-positive result P(T+) = P(T + |D+) x P(D+) + P(T + |D−) x P(D−) in dependence of sensitivity, specificity, and prevalence (here P = probability, D+/D− = SSD positive/negative, and T+/ T− = test positive/negative) to show that there is an issue that goes beyond our (possibly even selectively distorted) case studies. We considered a scenario ‘incompatible’ with our study findings if the expected proportion of test positives was below or above the boundaries of the 99%CI of the proportion of test positives observed in our data. Ratios of expected test-positive results / observed test-positive results were calculated to compare deviations as a function of pretest probability; ratios of expected test-positive prevalence / assumed true prevalence were calculated to compare expected distorted prevalence estimates. Additionally, we simulated sensitivities and specificities to identify values that would be compatible with our proportion of test positives (see Supplementary material). To allow readers to reproduce and expand our calculations, the Supplementary material also contains a respective calculation template.

## 3 Results

### 3.1 Characteristics of participants and basic psychometric findings

Of the 501 participants included in the original study, 81 were excluded from the present analysis due to missing sum scores for the PHQ-15 (*n* = 76) and/or SSD-12 (*n* = 39). Among the 420 patients whose data were analyzed, 53% were women, and the mean age (± SD) was 45 ± 16 years (see Table 1). The most frequent reasons for primary care encounters were medical procedures (blood tests, etc., 22%) and musculoskeletal (21%) and respiratory (20%) problems. The median (IQR) of the sum score was 6 (3 to 9) for the PHQ-15 and 10 (4 to 16) for the SSD-12.

**Table 1:** Characteristics of participants (*n* = 420).

Variable (missing values)	Absolute frequency (percentage) or mean ± SD
Female (0)	222 (53%)
Age in years (0)	45 ± 16
High school graduation (1)	130 (31%)
Private insurance (2)	46 (11%)
At least one chronic disease (1)	151 (36%)
Without appointment (0)	208 (50%)
*Reasons for encounter according to ICPC-2 categories (0)*	
Procedures	92 (22%)
Musculoskeletal	90 (21%)
Respiratory	82 (20%)
Digestive	35 (8%)
Neurologic	21 (5%)
Cardiovascular	18 (4%)
Psychological	11 (3%)
*Most frequent single ICPC-2 codes (0)*	
Upper respiratory infection acute	64 (15%)
Blood test	20 (5%)
Neck symptoms/ complaints	17 (4%)
Preventive immunization/medication	17 (4%)
Headache	13 (3%)
Lower back symptoms/complaints	12 (3%)
*PHQ-15*	
Mean (standard deviation)	6.8 ± 5.0
Median / IQR / Range	6 / 3–9 / 0–26
*SSD-12*	
Mean (standard deviation)	11.2 ± 9.0
Median / IQR / Range	10 /4–16 / 0–43

ICPC-2, International Classification of Primary Care, second version; IQR, interquartile range.

### 3.2 Proportion of test positives based on the different cut-off combinations

Using the cut-off combination #1 (≥9 and ≥ 23), 38 of our study participants (9, 99%CI, 6 to 13%) were classified as test positives for SSD (Table 2). The number of participants classified as test positive increased to 98 (23, 99%CI, 18 to 29%) when applying the cut-off combination #2 (≥8 and ≥ 13).

**Table 2:** Test results in screening for SSD using the PHQ-15 and the SSD-12 in our primary care sample at cut-off combination #1 recommended by Toussaint et al. (9) and cut-off combination #2 recommended by Cao et al. (10).

Using the cut-off combination #1 recommended by Toussaint et al.: ≥9 / ≥23	SSD-12 ≥ 23	SSD-12 < 23	Sum
PHQ-15 ≥ 9	**38 (9%)**	79 (19%)	117 (28%)
PHQ-15 < 9	13 (3%)	290 (69%)	303 (72%)
Total	51 (12%)	369 (88%)	420 (100%)

Using the cut-off combination #2 recommended by Cao et al.: ≥8 / ≥13	SSD-12 ≥ 13	SSD-12 < 13	Sum
PHQ-15 ≥ 8	**98 (23%)**	59 (14%)	263 (63%)
PHQ-15 < 8	58 (14%)	205 (49%)	157 (37%)
Total	156 (37%)	264 (63%)	420 (100%)

### 3.3 Comparison of observed and expected test-positive findings

The sensitivity and specificity estimates for combination #1 reported by Toussaint et al. were incompatible with the number of test-positive findings in our sample (9% for combination #1) regardless of the, respectively, assumed true prevalence level (see Table 3). For example, if the true prevalence of SSD in our sample was 15%, we would have expected 36% test-positive findings, i.e., if the sensitivity and specificity estimates would be applicable to our unselected primary care patients. Based on the law of total probability, the proportion of test-positive participants in our group could not be below 30%, irrespective of the actual SSD prevalence, as P(T+) = 0.69 x P(D+) + (1–0.70) x (1 - P(D+)) = 0.30 + 0.39 x P(D). The frequency of positive test results observed in our study is only plausible if one assumes a lower sensitivity and a much higher specificity (outside the upper limit of the 95% confidence interval for the estimate reported by Toussaint et al.; see Supplementary material). For example, if the sensitivity was 0.45 and specificity 0.97, our observed test-positive frequency of 9% would be expected if true SSD prevalence was 15% (see Table 4 for the example and Supplementary material for further scenarios).

**Table 3:** Expected test results in a sample of 420 participants when using the sensitivity and specificity estimates reported by Toussaint et al. (10) and Cao et al. (11).

Scenario	Observed estimates in Toussaint or Cao		Expected test findings based on the assumed true prevalence and sensitivity and specificity					*n* expected test positive/*n* observed test positives	% expected test positive/% assumed true prevalence
Assumed true prevalence	Sensitivity specificity		*n* true positive	*n* false positive	*n* false negative	*n* true negative	*n* test positive (%)		
**#**1 Cut-offs recommended by Toussaint et al.: ≥9/≥23 resulting in 38 (9%; 99% CI 6, 13%) observed test positives in our study									
1%	0.69	0.70	3	125	1	291	**128 (30.4%)**	3.4	30.4
5%			14	120	7	279	**134 (32.0%)**	3.5	6.4
10%			29	113	13	65	**142 (33.9%)**	3.7	3.4
15%			43	107	20	250	**151 (35.9%)**	4.2	2.4
20%			58	101	26	235	**159 (37.8%)**	4.4	1.9
25%			72	95	33	221	**167 (39.8%)**	4.6	1.4
30%			87	88	39	206	**165 (41.7%)**	4.8	1.2
40%			116	52	76	176	**192 (45.6%)**	5.0	1.1
#2 Cut-offs recommended by Cao et al.: ≥8/≥13 resulting in 98 (23%; 99% CI 18, 29%) observed test positives in our study									
1%	0.68	0.80	3	83	1	333	86 (20.5%)	0.9	20.5
5%			14	80	7	319	94 (22.4%)	1.0	4.5
10%			29	76	13	302	105 (24.8%)	1.1	2.5
15%			43	71	20	286	114 (27.2%)	1.2	1.8
20%			57	67	27	269	**124 (29.6%)**	1.3	1.5
25%			71	63	34	252	**134 (32.0%)**	1.4	1.3
30%			86	59	40	235	**145 (34.4%)**	1.5	1.1
40%			114	54	50	202	**165 (39.2%)**	1.7	1.0

Expected test results are shown, assuming a wide range of true prevalence expected screening results in a sample of 420 participants. Values are absolute numbers and proportions (percentages). Bold figures indicate that the number of expected test positives is incompatible with the number of observed test positives in our sample as it is not covered by the 99%CI of the latter. Minor deviations in numbers due to rounding.

**Table 4:** Expected test results in a sample of 420 participants when using the sensitivity and specificity estimates fitting well with the frequency of test-positive findings observed in our study.

Scenario	Observed estimates in Toussaint or Cao		Expected test findings based on the assumed true prevalence and sensitivity and specificity					*n* expected test positive/*n* observed test positives	% expected test positive/% assumed true prevalence
Assumed true prevalence	Sensitivity	Specificity	*n* true positive	*n* false positive	*n* false negative	*n* true negative	n test positive (%)		
#1 Cut-offs recommended by Toussaint et al.: ≥9/≥23 resulting in 38 (9%; 99% CI 6%; 13%) observed test positives in our study									
1%	0.45	0.97	2	12	2	403	**14 (3.4%)**	0.4	3.4
5%			9	12	13	387	**21 (5.1%)**	0.6	1.0
10%			19	11	23	367	30 (7.2%)	0.8	0.7
15%			28	11	35	346	39 (9.3%)	1.0	0.6
20%			38	10	46	326	48 (11.4%)	1.3	0.6
25%			47	9	58	306	**57 (13.5%)**	1.5	0.5
30%			57	9	69	285	**66 (15.6%)**	1.7	0.5
40%			76	8	92	244	**83 (19.8%)**	2.2	0.5
#2 Cut-offs recommended by Cao et al.: ≥8/≥13 resulting in 98 (23%; 99% CI 18%; 29%) observed test positives in our study									
1%	0.60	0.85	3	62	2	353	**65 (15.4%)**	0.7	15.5
5%			13	60	8	339	**72 (17.3%)**	0.7	3.5
10%			25	57	17	321	82 (19.5%)	0.8	2.0
15%			38	54	25	303	91 (21.8%)	0.9	1.5
20%			50	50	34	286	101 (24.0%)	1.0	1.2
25%			63	47	42	268	119 (26.3%)	1.1	1.1
30%			76	44	50	250	120 (28.5%)	1.2	1.0
40%			101	38	67	214	**139 (33.0%)**	1.5	0.8

Minor deviations in numbers due to rounding.

The sensitivity and specificity estimates for combination #2 reported by Cao et al. were compatible with the number of test-positive findings in our sample (23% for combination #2) regardless of whether a true SSD prevalence of 15% or less is assumed. Yet, if a true SSD prevalence of 15% is assumed, slightly lower sensitivity (60%) and slightly higher specificity (85%) would be more plausible in comparison with Cao et al. (11).

### 3.4 Over- and under-estimation of prevalence

If the sensitivity and specificity estimates reported by Toussaint et al. and Cao et al. were applied to unselected primary care patients, the expected proportion of test-positive findings would overestimate true prevalence unless it is very high (see the last column in Table 3). Instead, if the lower sensitivity and higher specificity values fitting with the findings of our study are used, true prevalence would be underestimated when using the cut-off combination #1 if the true prevalence of SSD was above 5% (see the last column in Table 4). In the case of the cut-off combination #2, relevant overestimation would occur in the case of a true prevalence of up to 15%. If the actual prevalence were 20% or even higher, the expected proportion of positive tests would roughly correspond to the actual prevalence.

## 4 Discussion

Applying the cut-off value combinations recommended by the published diagnostic accuracy studies performed in high-prevalence settings of SSD to our unselected primary care patients resulted in two very different proportions of participants who tested positive in SSD by the self-report questionnaires PHQ-15 and SSD-12 (9 and 23%, respectively). The sensitivity and specificity estimates found by Toussaint et al. for combination #1 were incompatible with our findings, and the estimates found by Cao et al. for combination #2 were partly compatible.

Our study was not a diagnostic accuracy study comparing questionnaire findings with diagnoses based on validated semi-structured or structured interviews as reference standards. Therefore, the true prevalence of SSD among our study participants remains unknown, and we could not directly investigate sensitivity and specificity for defined cut-off values. However, rigorous prevalence and diagnostic accuracy studies for SSD in primary care are still lacking. Recent scoping reviews (6, 13) found that available prevalence estimates for SSD in primary care or the general population are based on proxy diagnoses based on self-report questionnaires. Other studies [e.g. (14)] also use questionnaire combinations to split participants into those with and without suspected SSD. In this situation, the strong inconsistencies in cut-offs identified in our analyzes can provide some important messages to researchers and healthcare professionals.

First, our analyzes strongly suggest that the sensitivity and specificity found by Toussaint et al. (10) for their recommended cut-off combination to detect SSD in psychosomatic outpatients are not applicable to unselected primary care patients. It seems likely that in such a setting, specificity is much higher and sensitivity lower. A higher specificity not only means a higher probability that a person testing negative does not have SSD but also that a positive result makes the presence of SSD more likely compared to a lower value. Lower sensitivity means that fewer persons who actually have SSD will be tested positive, and more patients will be false negative. It is sometimes thought that sensitivity and specificity are “stable” characteristics of a test (15). However, there are many examples showing that these parameters vary between different populations (16–20). The term ‘spectrum effect’ is used if true differences in the distribution of disease (severity) or prior diagnostic testing are the reasons for variations in diagnostic accuracy in different populations (17). It has been shown that specificity can increase while sensitivity decreases if many of the study participants score far below the applied cut-off values (18). On the other hand, sensitivity can increase while specificity decreases if previous diagnostic investigations lead to a selection of patients with more severe symptoms (21, 22). Our findings suggest that the spectrum effect might be large in the case of SSD when comparing psychosomatic outpatients and unselected primary care patients.

Second, our findings also suggest that the cut-off combination #1 is too strict to adequately balance sensitivity and specificity values in unselected primary care patients. An SSD-12 score of ≥23 makes a true diagnosis of SSD very likely (when assuming a true prevalence between 10 and 25%), but many individuals with SSD that score below 23 may be missed. Whether combination #2 with much lower cut-offs is a good choice for primary care remains unclear. In a recent re-analysis of their data (22), Cao et al. discuss a slightly higher cut-off (≥16) when applying the SSD-12 only, without the PHQ-15.

Third, our analyzes show that it is almost impossible at the current state to assess whether prevalence estimates for SSD in primary care based on proxy diagnoses from self-report questionnaires are accurate. There is a possibility of (gross) overestimates or underestimates. Given the example of major depression, an individual patient data meta-analysis of 44 studies on diagnostic accuracy found that the widely used Patient Health Questionnaire-9 (PHQ-9) identifies 2.5 times as many major depression cases as a validated semi-structured diagnostic interview (23). Bayesian approaches have been proposed to estimate prevalence, sensitivity, and specificity in studies without diagnostic interviews, taking into account prior information from diagnostic studies (24). Recently, a Bayesian latent class model was used to account for imperfect diagnostic accuracy in the interpretation of results from a large survey in 27 European countries using the PHQ-8 (a shortened version of the PHQ-9). Based on the PHQ-8 alone, this study estimated an overall prevalence of major depression of 6.4%. Taking into account the diagnostic misclassification, the study found a much lower prevalence of 2.1% (25). This could be a similar problem to our hypothesis regarding a misjudgment of SSD. However, as only two diagnostic studies with two very different cut-off recommendations are available, it may be too early to conduct such analyzes to estimate the prevalence of SSD. Our approach is descriptive, non-inferential, and easily applicable for researchers who want to roughly check whether the results of their questionnaire survey are consistent with published estimates of diagnostic accuracy. However, when researchers apply our approach, they must be aware that both sensitivity, specificity and estimated expected frequencies are all subject to uncertainty.

Despite the different cut-off combinations recommended by Toussaint et al. (10) and Cao et al. (11), the high estimates found for the area under the curve in both studies suggest that the combination of PHQ-15 and SSD-12 show good diagnostic accuracy in terms of detecting patients at risk for SSD. Particularly in primary care, it also could be considered using the SSD-12 alone. The area under the curves in available analyzes (11, 12) suggests satisfactory to good accuracy. Furthermore, in a recent study of primary care patients, SSS-8 scores (a shorter version of the PHQ-15) were found to be more influenced by symptoms of uncomplicated acute infections than SSD-12 scores (26). In any case, setting specific cut-off values for self-report questionnaires covering the A and B criteria of the SSD may be needed, especially for research purposes. For clinical use, it is probably more useful to interpret actual questionnaire score values together with other clinical information available instead of dichotomizing the questionnaire findings into SSD positive and SSD negative. In general, the PHQ-15 and SSD-12 values can be valuable for general practitioners to identify patients who need psychological help due to strong concerns about their persistent somatic symptoms. Obviously, the PHQ-15 and SSD-12 alone are not sufficient to make a diagnosis of SSD, but they give solid indications of the presence of SSD.

Our study was conducted in a limited number of practices in one region of Bavaria. As the primary aim of the original study was to investigate differences between patients with and without appointments, our study sample is probably not fully representative of all patients seeking care in the study practices. Patients without appointments were slightly younger and more often had acute complaints than patients with appointments. However, results regarding SSD were almost identical in both groups. Furthermore, our case mix and mean age compare well with a large study of consecutive primary care patients in Germany (27). Therefore, although our study participants may not be fully representative of unselected primary care patients in Germany, it seems very unlikely that our results are subject to major bias regarding the applicability of the recommended cut-off scores.

## 5 Conclusion

Our analyzes strongly suggest that the sensitivity and specificity found by Toussaint et al. (10) for their recommended cut-off combination #1 in psychosomatic outpatients are not applicable to unselected primary care patients and that the cut-off for the SSD-12 (≥23) seems too high. The cut-off combination #2 recommended by Cao et al. (11) seems more applicable. Prevalence estimates for SSD based on proxy diagnoses from self-report questionnaires using cut-off recommendations derived from other clinical settings have a risk of resulting in incorrect classifications in primary care. Studies that compare screening findings by questionnaires with validated diagnostic interviews as reference standards in primary care populations are urgently needed in order to make statements on the accuracy of the scales to detect patients at risk for SSD.

## Data availability statement

The original contributions presented in the study are included in the article/Supplementary material. Further inquiries can be directed to the corresponding author.

## Ethics statement

The studies involving humans were approved by Ethikkommission Technical University Munich. The studies were conducted in accordance with the local legislation and institutional requirements. Written informed consent for participation was not required from the participants or the participants’ legal guardians/next of kin because due to anonymised data collection, oral consent was obtained.

## Author contributions

VS: Conceptualization, Data curation, Formal analysis, Writing – original draft, Writing – review & editing. AT: Supervision, Writing – review & editing. AH: Conceptualization, Formal analysis, Methodology, Writing – review & editing, Writing – original draft. CT: Writing – review & editing. BR: Project administration, Writing – review & editing. PH: Supervision, Writing – review & editing. JG: Supervision, Writing – review & editing. AS: Funding acquisition, Methodology, Project administration, Supervision, Writing – review & editing. KL: Conceptualization, Investigation, Methodology, Supervision, Writing – original draft, Writing – review & editing.

## Group members of the POKAL-Study Group

The POKAL-Study-Group [PrädiktOren und Klinische Ergebnisse bei depressiven ErkrAnkungen in der hausärztLichen Versorgung (POKAL, DFG-GRK 2621)] consists of the following principal investigators: Tobias Dreischulte, Peter Falkai, Jochen Gensichen, Peter Henningsen, Markus Bühner, Caroline Jung-Sievers, Helmut Krcmar, Karoline Lukaschek, Gabriele Pitschel-Walz, and Antonius Schneider. The following doctoral students are also members of the POKAL-Study-Group: Jochen Vukas, Puya Younesi, Feyza Gökce, Victoria von Schrottenberg, Petra Schönweger, Hannah Schillock, Jonas Raub, Philipp Reindl-Spanner, Lisa Hattenkofer, Lukas Kaupe, Carolin Haas, Julia Eder, Vita Brisnik, Constantin Brand, Chris Ebert, Marie Emilia Vogel, Clara Teusen, and Katharina Biersack.
